# Supplementary material for: Content-rich biological network constructed by mining PubMed abstracts
Source: BMC Bioinformatics. 2004 Oct 8;5:147. doi: 10.1186/1471-2105-5-147 (PMC528731; doi:10.1186/1471-2105-5-147)
Supplement: Additional File 2 — The original results of the above study (non-essential files are deleted to keep the file size under the limit set by BMC bioinformatics). [file 1471-2105-5-147-S2.bz2 › chilibotAdditionalFile2/dip20/113ID8645696E457/html/ATPA_ATPH.html]

 


 **ATPA** and **ATPH** 
  
Found 15 abstracts in PubMed, retrieved 15.  
 

 What does Google say? 
 PDF only 
| .edu only 

---

**Interactive relationship** (e.g. stimulation, inhibition, etc)

**Neutral relationship**- 8 762 4766 indicate that primarily ribosomes reinitiating after termination on  **atpH**  are responsible for inducing refolding of the  **atpA**  TIR.  Ref: 7517937 J Biol Chem, 1994
- The gene pair atpHA is tightly coupled, whereby  **atpA**  is translated at least three times more efficiently than  **atpH** .  Ref: 7517937 J Biol Chem, 1994
- The results indicate that in the E. coli atp operon, translation directed by the atpB,  **atpH**  and atpG TIRs is less efficient than that directed by the  **atpA**  TIR, and are thus consistent with earlier measurements of direct atp gene expression.  Ref: 2529415 Mol Microbiol, 1989
- This paper describes the  **atpA**  gene cluster in the C. reinhardtii chloroplast genome, which includes the  **atpA** , psbI, cemA, and  **atpH**  genes, encoding the alpha subunit of the coupling factor 1 CF1 ATP synthase, a small photosystem II polypeptide, a chloroplast envelope membrane protein, and subunit III  [ **ATPH** ]  of the CF0 ATP synthase, respectively.  Ref: 9625716 Plant Physiol, 1998
- However, there is no fixed stoichiometry of coupling mutations in  **atpH**  lead to increases in the translation ratio  **atpA**   **atpH**  of up to approximately 40 fold.  Ref: 7517937 J Biol Chem, 1994

**Non-interactive relationship** (e.g. studied together, co-existance, homology, etc.)

- The precise start positions of the atpE c, atpF b,  **atpH**  delta and  **atpA**  alpha genes have been defined by comparison of the potential coding sequences with the known amino acid sequence of the c subunit and the determined N terminal amino acid sequences of the respective subunits.  Ref: 6278247 Mol Gen Genet, 1981
- The 5 and 3 transcribed flanking regions of  **atpA**  and  **atpH**  from maize, wheat and tobacco are conserved in size, but contain few putative regulatory elements which are conserved either in their spatial arrangement or sequence complexity.  Ref: 2885245 Genetics, 1987
- Five genes, atpE, atpF,  **atpH** ,  **atpA**  and atpG, were shown to be translationally coupled to various degrees of tightness.  Ref: 1830358 Mol Microbiol, 1991
- We show that promoters precede the  **atpA** , psbI, and  **atpH**  genes, but not the cemA gene,.  Ref: 9625716 Plant Physiol, 1998
- Their order counterclockwise is atpB a, atpE c, atpF b,  **atpH**  delta and  **atpA**  alpha.  Ref: 6278247 Mol Gen Genet, 1981
- The sequence starts from one end JLA of the large single copy region and encompasses genes for 21 tRNAs, six ATPase subunits  **atpA** , atpB, atpE, atpF,  **atpH**  and atpI, two photosystem I polypeptides psaA and psaB, four photosystem II polypeptides psbA, psbC, psbD and psbG, five ribosomal proteins rps2, rps4, rps7, rps 12 and rps14, and three RNA polymerase subunits rpoB, rpoC1 and rpoC2.  Ref: 2974085 J Mol Biol, 1988
- The translational initiation rates directed by the translational initiation regions TIRs of the atpB,  **atpH** ,  **atpA**  and atpG genes of Escherichia coli were investigated using lacZ fusions present on plasmids as well as integrated into the chromosome.  Ref: 2529415 Mol Microbiol, 1989
- Individual subunits of ATP synthase, encoded by the eight genes of the atp operon  **atpA**  through  **atpH** , have been found to be synthesized at a 10 fold range in molar amounts D.L.  Ref: 7690747 J Bacteriol, 1993
- No genes for photosynthetic functions have been found, except for sequences encoding six subunits of the ATP synthase  **atpA** , atpB, atpE, atpF,  **atpH** , and atpI.  Ref: 12111556 Mol Genet Genomics, 2002
- The C. thermoaceticum atp operon contained nine genes arranged in the order atpI i, atpB a, atpE c, atpF b,  **atpH**  delta,  **atpA**  alpha, atpG gamma, atpD beta, and atpC epsilon.  Ref: 9171425 J Bacteriol, 1997
- Eight genes atpI, atpB, atpE 1, atpE 2, atpE 3, atpF,  **atpH** , and  **atpA**  upstream of and contiguous with the previously described genes atpG, atpD, and atpC were cloned from chromosomal DNA of Acetobacterium woodii.  Ref: 10567365 J Biol Chem, 1999
- The nucleotide sequences of the maize plastid genes for the alpha subunit of CF1  **atpA**  and the proteolipid subunit of CF0  **atpH**  are presented.  Ref: 2885245 Genetics, 1987
- There is particularly tight coupling between  **atpH**  and  **atpA** .  Ref: 2529415 Mol Microbiol, 1989
- The linear order of these genes, 5 rps2 atpI  **atpH**  atpF  **atpA**  rps18 3, encoding ribosomal protein S2, chloroplast ATP synthase subunits CF0IV, CF0III, CF0I, CF1 alpha and ribosomal protein S18, respectively, is similar to the equivalent operons of prokaryotes, cyanelles and land plant chloroplasts.  Ref: 8435857 Curr Genet, 1993
- These fragments contain six closely linked reading frames that were identified as atpI,  **atpH** , atpG, atpF, atpD, and  **atpA** , coding for subunits IV, III, II, I, delta, and alpha, respectively.  Ref: 1532839 J Mol Biol, 1992
- The regions of the spinach and pea chloroplast genomes containing the ATP synthase genes  **atpA** , atpF and  **atpH**  have been sequenced.  Ref: 2443718 J Mol Biol, 1987
- Molecular evolution and nucleotide sequences of the maize plastid genes for the alpha subunit of CF1  **atpA**  and the proteolipid subunit of CF0  **atpH** .  Ref: 2885245 Genetics, 1987
